# CT lymphography for sentinel lymph node mapping of clinically N0 early oral cancer

**DOI:** 10.1186/s40644-019-0258-9

**Published:** 2019-11-12

**Authors:** Satomi Sugiyama, Toshinori Iwai, Toshiharu Izumi, Keita Ishiguro, Junichi Baba, Senri Oguri, Kenji Mitsudo

**Affiliations:** 1 Department of Oral and Maxillofacial Surgery/Orthodontics, Yokohama City University hospital, 3-9 Fukuura, Kanazawa-ku, Yokohama, Kanagawa 236-0004 Japan; 2 Department of Radiology, Yokohama City University Hospital, 3-9 Fukuura, Kanazawa-ku, Yokohama, Kanagawa 236-0004 Japan

**Keywords:** Early oral cancer, Sentinel lymph node, Metastasis, Computed tomography lymphography

## Abstract

**Background:**

The objectives of this retrospective study were to evaluate the usefulness of computed tomography lymphography (CTL) and to clarify the optimal timing of CTL in sentinel lymph node (SLN) mapping of clinically N0 early oral cancer.

**Methods:**

Twenty patients with clinically N0 early oral cancer underwent CTL with a 128 multi-detector row CT scanner to detect SLN the day before resection of primary tumor and SLN biopsy with indocyanine green (ICG) fluorescence guidance. CT scanning was performed in the first 10 patients at 2, 5, and 10 min after submucosal injection of iopamidol and in the remaining 10 patients at 2, 3.5, 5, and 10 min after the injection of contrast medium. We evaluated the SLN detection rate at each scan timing and the number and location of SLNs. We evaluated whether CTL-enhanced SLNs could be identified intraoperatively as ICG fluorescent lymph nodes.

**Results:**

SLNs were detected by CTL in 19 of the 20 patients (95.0%), and the mean number of SLNs was 2 (range, 1–4). All SLNs were located on the ipsilateral side; 35 of 37 SLNs were located at level I and II, and 2 SLNs were lingual lymph nodes. All SLNs could be detected 2 min and 3.5–5 min after contrast medium injection, and CTL-enhanced SLNs could be identified intraoperatively as fluorescent lymph nodes.

**Conclusions:**

CTL could facilitate the detection of SLNs in early oral cancer, and the optimal timing of CT scanning was at 2 and 5 min after injection of contrast medium.

## Background

Cervical lymph node metastasis is an important prognostic factor in oral cancer. Because occult metastasis is found during observation after primary surgery in 20 to 30% of patients with N0 early oral cancer [1], some studies recommend prophylactic neck dissection (ND). Nonetheless, 70 to 80% of patients with early oral cancer who underwent ND may experience complications such as facial nerve paralysis or shoulder dysfunction. Therefore, the concept of the sentinel lymph node (SLN), which is the first lymph node to receive drainage from a primary tumor, has recently been applied in detecting early lymph node metastasis in oral cancer patients [1–12], and SLN biopsy (SLNB) has been demonstrated to be less invasive than prophylactic ND. Although SLN detection using radioisotope (RI) tracers is commonly performed [4, 5, 8–14], RI cannot be used in certain institutions due to restrictions regarding the handling of radioactive material [2]. Furthermore, SLN detection is difficult when the SLN is close to the site of RI injection, due to shine-through artifacts [2]. Another method for visualizing SLNs and lymphatics is computed tomography lymphography (CTL), which does not require special equipment and has recently been applied in melanoma, breast, esophageal, and gastric cancer [15–21]. However, there are few reports of CTL for SLN mapping in patients with N0 early oral cancer [2, 22, 23]. The objectives of this retrospective study were to evaluate the usefulness of CTL and to clarify the optimal scan timing of CTL in SLN mapping of N0 early oral cancer.

## Materials and methods

A total of 20 early oral cancer patients without cervical lymph node metastasis were enrolled in our study between June 2016 and June 2018. All patients had primary cancer without prior surgery, chemotherapy, and/or radiotherapy. The primary cancer and cervical lymph nodes before surgery were assessed using 4 modalities: enhanced-computed tomography (CT), magnetic resonance imaging (MRI), 18F-fluorodeoxyglucose positron emission tomography/computed tomography (FDG-PET/CT), and ultrasonography (US). The criteria for a diagnosis of N0 oral cancer at our institutions can be summarized as follows: (i) minimum axial diameter of the node < 10 mm and no rim enhancement on enhanced CT; (ii) maximum standardized uptake < 2.5 on PET/CT; and (iii) presence of hilar echoes on US. TNM staging was classified according to the Union for International Cancer Control staging system (7th edition). This retrospective study was approved by the institutional review board of our university and was conducted according to the Declaration of Helsinki. Written informed consent was obtained from all patients.

CTL was performed with a 128 multi-detector row CT scanner (Siemens SOMATOM Definition AS+; Siemens Healthcare GmbH, Erlangen, Germany) to detect SLNs the day before resection of the primary tumor and SLNB. Patients were placed in the supine position and CT scanning was performed with the following parameters: tube voltage of 80 kVp, 400 mAs, helical thickness of 0.6 mm, field of view of 220 mm, and rotation time of 1.0 s. First, non-contrast CT images of the oral cavity and neck were obtained. After local anesthesia, a total of 2.0 mL of iopamidol (Iopamiron 300; Bayer Yakuhin Co., Ltd., Osaka, Japan) was injected submucosally into 4 points around the tumor with a 27-gauge needle and the injected sites were massaged. CT scanning was performed in the first 10 patients at 2, 5, and 10 min after administration of iopamidol. Furthermore, additional CT scanning at 3.5 min after injection of contrast medium was performed in the remaining 10 patients to clarify more optimal scanning time based on results of first 10 patients. SLNs were identified as the first enhancing lymph node in the lymphatic flow from the sites of injection of the contrast medium, and the CT images with three-dimensional (3D) reconstruction were analyzed on the day of the procedure. The 3D reconstruction was performed using the cinematic volume rendering technique with syngo.via imaging software (Siemens Healthcare GmbH, Erlangen, Germany).

SLNB was performed under indocyanine green (ICG) fluorescence guidance 1 day after SLN mapping of CTL. A total of 2.0 mL (5 mg/mL) ICG (Diagnogreen 0.5%; Daiichi-Sankyo Co., Ltd., Tokyo, Japan) was injected into each of 4 points around the tumor, and the HyperEye Medical System (Mizuho Co., Ltd., Tokyo, Japan) was used intraoperatively to detect SLNs that were mapped by CTL [7]. It was evaluated whether SLNs enhanced by CTL could be identified intraoperatively as ICG fluorescent lymph nodes.

## Results

The characteristics of the patients are summarized in Additional file 1: Table S1. There were 12 men and 8 women with a median age of 66 (range 23–86) years. Body mass index (BMI) was 17.5–32.0. The most common primary tumor site was the tongue, and only 2 of the 20 patients had cancer of floor of the mouth. In total, 15 patients (75.0%) had T1 and 5 patients had T2 (25.0%) disease.

**Fig. 1:**
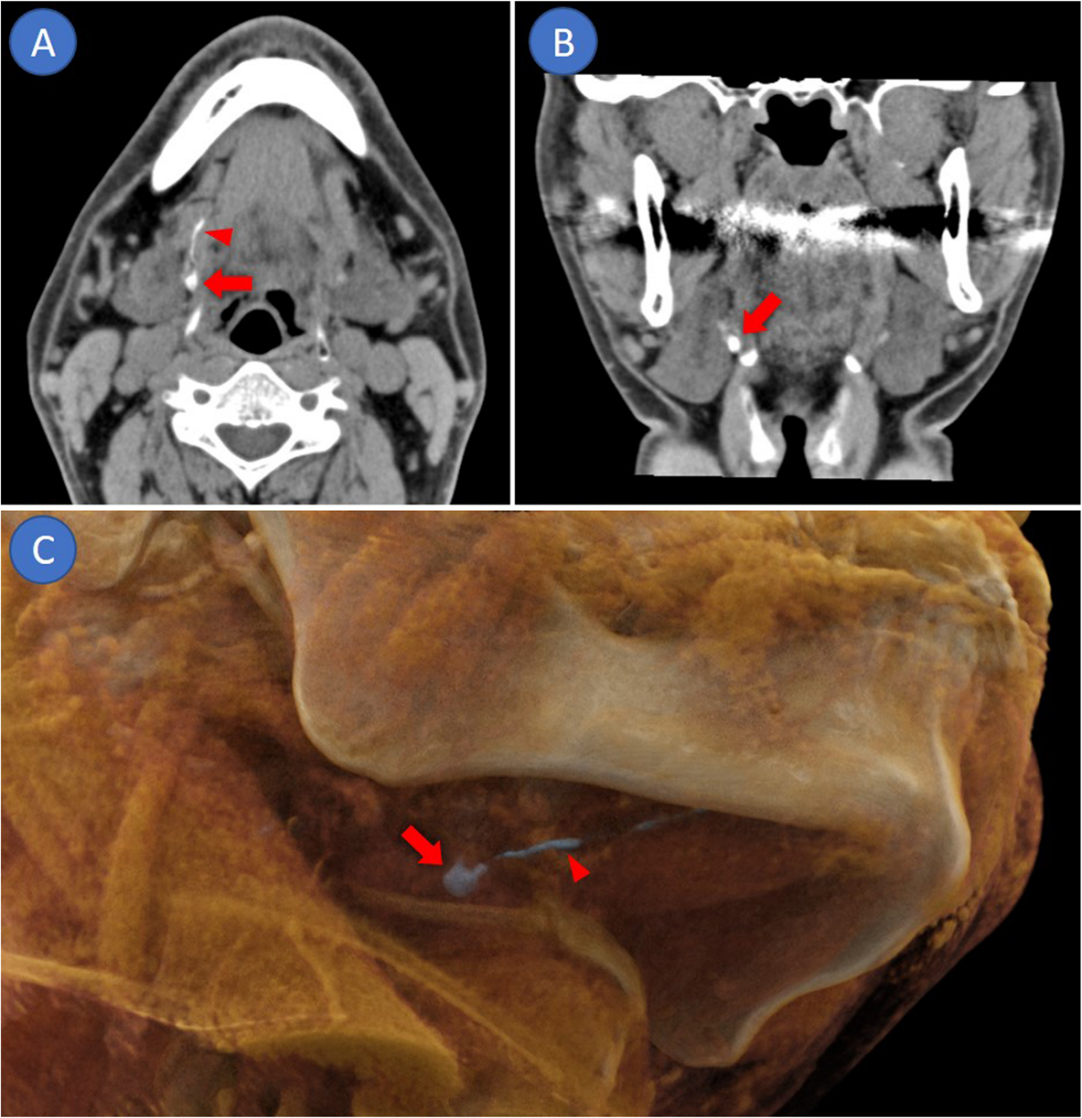
CT lymphography. **a**: Axial image, **b**: coronal image, **c**: 3D image with cinematic volume rendering technique. Arrows indicate sentinel lymph node. Arrowheads indicate lymphatics

**Fig. 2:**
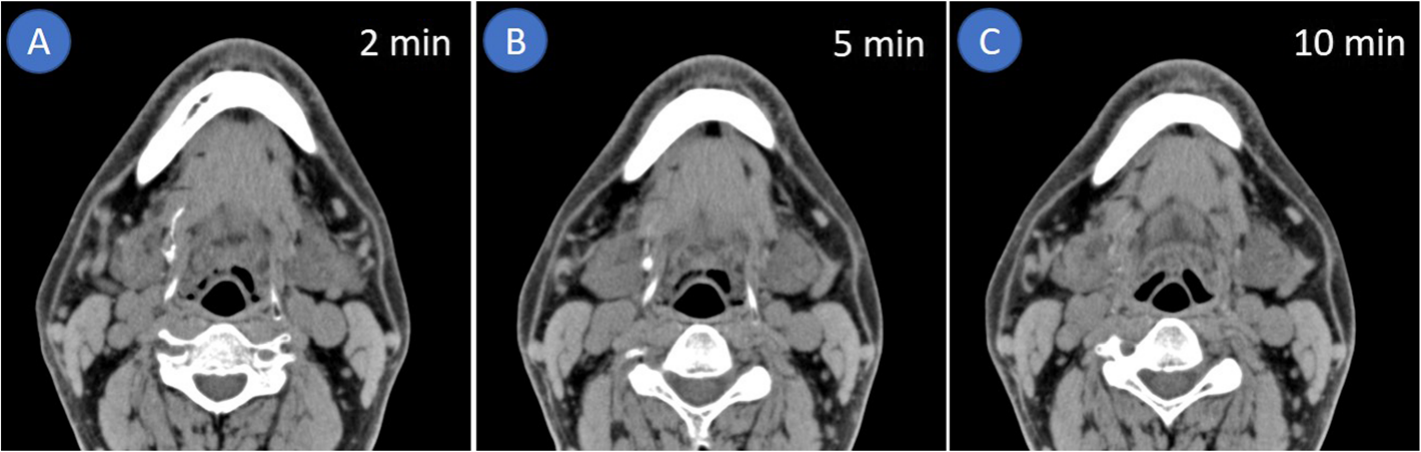
CT lymphography (axial image). **a**: Sentinel lymph node and lymphatic visualized 2 min after local injection of iopamidol. **b**: Sentinel lymph node clearly enhanced 5 min after contrast injection, with disappearance of the lymphatic vessel. **c**: Disappearance of both the sentinel lymph node and lymphatic vessel 10 min after contrast injection

SLNs were detected by CTL in 19 of the 20 patients (95.0%) (Fig. 1; Table 1), and neither SLN nor lymphatic vessel draining from the iopamidol injection site of was detected in 1 patient (Patient K). Although 18 of 19 patients (94.7%) had both SLNs and lymphatics, SLN only was detected in 1 patient. The number of SLNs was 0 in 1 patient, (5.0%), 1 in 6 patients (30.0%), 2 in 9 patients (50.0%), 3 in 3 patients (15.0%), and 4 in 1 patient (5.0%). The total number and mean number of SLNs were 37 and 1.9, respectively. All SLNs were located on the ipsilateral side; 17 (45.9%) of 37 SLNs were located at level IIA, 15 (40.5%) at level IB, 2 (5.4%) at level IA, and 1 (2.7%) at level IIB. Two SLNs (5.4%) were lingual lymph nodes. In 19 SLNs in the first 10 patients, the detection rates at 2, 5, and 10 min after the contrast medium injection were 73.7, 78.9, and 42.1%, respectively (Fig. 2). The first enhancement of SLNs was found in 14 lymph nodes (83.3%) at 2 min after the injection, and CTL at 5 min after contrast injection showed enhancement of the remaining 5 nodes (16.7%). All SLNs could be detected by CTL both 2 and 5 min after contrast injection. In patient G, SLN enhancement was observed on CTL at 2 min after contrast injection but was washed away on CTL at 5 min after. In patients C and H, some SLNs were enhanced only 5 min after contrast injection. In 18 SLNs of the remaining 10 patients with additional CT scanning, the detection rates of SLNs at 2, 3.5, 5, and 10 min after contrast injection were 88.9, 94.4, 94.4, and 22.2%, respectively. SLN enhancement was found in 16 lymph nodes (88.9%) at 2 min after contrast injection, and CTL 3.5 and 5 min after contrast injection showed enhancement of the remaining 2 lymph nodes (11.1%). In some cases (Patients D, H, Q, and R), SLNs were detected at different scan timings. All SLNs enhanced by CTL could be identified intraoperatively as ICG fluorescent lymph nodes.

**Table 1:** Results of CTL in oral cancer patients

Patient code	SLN No.	SLN location	Detection of SLN			
			Scan timing of CTL			
			2 min	3.5 min	5 min	10 min
A	1	IIA	–	No scan	+	+
	2	IIA	–	No scan	+	+
B	3	IB	+	No scan	+	+
	4	IIA	+	No scan	+	–
	5	IIA	+	No scan	+	+
C	6	IA	–	No scan	+	–
D	7	IB	+	No scan	+	–
	8	IB	–	No scan	+	+
E	9	IB	+	No scan	+	+
F	10	IIA	+	No scan	+	+
G	11	IB	+	No scan	–	–
	12	IB	+	No scan	–	–
	13	IB	+	No scan	–	–
	14	IB	+	No scan	–	–
H	15	IB	+	No scan	+	–
	16	IB	–	No scan	+	–
I	17	IB	+	No scan	+	+
J	18	IIA	+	No scan	+	–
	19	IIA	+	No scan	+	–
K	–	–	–	–	–	–
L	20	IA	+	+	+	+
	21	IIA	+	+	+	–
	22	IIA	+	+	+	–
M	23	IIA	+	+	+	–
	24	IIA	+	+	+	–
N	25	IB	+	+	+	+
O	26	IB	+	–	–	–
	27	IIA	+	+	+	–
P	28	IIA	+	+	+	–
	29	IIA	+	+	+	–
	30	IIB	+	+	+	+
Q	31	Lingual lymph node	+	+	+	–
	32	IIA	–	+	+	–
R	33	Lingual lymph node	+	+	+	+
	34	IB	–	+	+	–
S	35	IA	+	+	+	–
	36	IIA	+	+	+	–
T	37	IB	+	+	+	–

CTL, computed tomography lymphography; SLN, sentinel lymph node

## Discussion

Because occult cervical lymph node metastasis during observation after primary surgery is found in 20 to 30% of patients with N0 early oral cancer and generally occurs at level I to III, supraomohyoid ND has been recommended [24, 25]. However, minimally invasive surgery such as SLNB is required to avoid the complications that can occur after ND, such as facial nerve paralysis. SLNB, which benefits patients without lymph node metastasis by avoiding unnecessary lymph node dissection, has been carried out in the treatment of various cancers such as melanoma, breast, and gastric cancer [15–21]. SLNB using RI is standard procedure and has been recently applied in oral cancer [9, 10, 14]. However, it cannot be performed in hospitals that do not have the required equipment and systems, thereby preventing its widespread use [1]. Furthermore, the gamma probe cannot provide visual information during surgery. An alternative to RI use, SLNB using ICG fluorescence imaging has been applied in N0 early oral cancer [6, 7, 26–28], but ICG fluorescent lymph nodes cannot always be detected transcutaneously, particularly when located deep (0.5–1.5 cm) in the subcutaneous tissue [7, 27]. Therefore, preoperative mapping of SLNs without RI is required for widespread application of SLNB under fluorescence guidance and the reliable detection of SLNs.

As an alternative method for preoperative visualization of SLNs and lymphatics, CTL does not require special equipment and has recently been applied in melanoma, breast, esophageal, and gastric cancer [15–20]. CTL has clarified, with high resolution, the detailed arrangement of lymphatics along the route in various malignant tumors when visualizing multiple lymph nodes. This then helped determine whether or not these were secondary lymph nodes or lymph nodes from separate channels, thus facilitating more accurate SLN identification [18]. Therefore, CTL without the shine-through effect is recommended for the accurate identification of SLNs and for better topographical 3D orientation before SLNB [18], and the reported detection rate of SLNs is 99 to 100% in breast cancer [15–17]. After SLN mapping using CTL, intraoperative fluorescent SLNB with ICG has been applied in breast cancer [15–17]. Although identification may become difficult following damage to the lymphatics by surgical manipulation, preoperative CTL with 3D reconstruction can help in identifying SLNs [18]. Therefore, we have also performed SLN mapping using CTL and SLNB under ICG fluorescence navigation for N0 early oral cancer as an alternative to RI use [7]. Because there are few reports of SLN mapping using CTL for N0 early oral cancer, the imaging protocol for CTL is not clear [2, 22, 23].

In a study of CTL for SLN mapping in patients with N0 early tongue cancer, Honda et al. [2, 23] injected 1.5 mL iopamidol (Iopamiron 370) into the peritumoral area. SLNs were detected by CTL in 88.9–90.3% of patients. The number of SLNs was 0 in 9.7–11.1% of patients, 1 in 35.5–44.4% of patients, 2 in 35.5–38.9% of patients, and 3 in 5.6–19.4% of patients (mean 1.6–1.8). In 28 patients, the detection rates of SLNs at 1, 3, 5, 10 min after contrast injection were 7.1, 53.6, 17.9, and 21.4%, respectively [2]. Their study required 4 separate CT scans for SLN mapping in tongue cancer patients. In contrast, in our CTL study involving the injection of 2 mL of Iopamiron 300 into the peritumoral area of early oral cancer patients, the SLN detection rate was higher at 95.0% and the mean number of SLNs was slightly higher 1.9 (range, 1–4). BMI of Patient K without SLN detection was low (17.5) and it seemed that BMI is not associated with SLN detection. SLNs could be identified by 2 separate CT scans (2 min and 3.5–5 min after contrast injection) and with lower radiation exposure. These discrepancies between the two studies may be attributed to the osmotic pressure gradient (about 4 for Iopamiron 370 and about 3 for Iopamiron 300) and the viscosity of the contrast medium (9.1 mPa·s and 4.4 mPa·s, respectively) as well as dose of the contrast agent.

The mean number of SLNs detected by lymphoscintigraphy or single-photon emission CT with CT (SPECT/CT) was 2 to 3 in N0 early oral cancer [5, 10, 12], and the most common location of SLNs was level I to III using RI [29]. Regarding the location of SLNs detected by CTL, Honda et al. [2] reported SLNs at level I to III; in our study, SLNs were at level I to II and in the sublingual region. Although metastasis to the sublingual lymph nodes is one of the crucial events in determining survival outcome in cancer of the tongue and floor of the mouth, few reports about the lingual lymph node are available because of its small size and close location to the primary tumor [22]. In 2012, Saito et al. [22] first reported that CTL revealed a lateral lingual lymph node as the SLN in a tongue cancer patient, and our study marks the second report in which SLN was detected in the sublingual region.

Lingual lymph nodes are classified as median and lateral [30], and the more common lateral lingual nodes, which might be the first echelon nodes in tongue cancer [31], are found on the lateral aspect of the genioglossus or on the hyoglossus muscle in direct relation to the sublingual glands. The lingual lymph nodes are not always present (incidence: 17.1 to 25.1%) [32–34], and lingual lymph node metastasis in oral cancer is rare [31, 33, 35–39] with a reported incidence of 2.1 to 14.3% [32, 33, 35]. Owing to the close proximity to the primary tumor and the frequency of extracapsular invasion, lingual lymph node metastasis can be misdiagnosed as local recurrence [38]. Therefore, lingual lymph node metastasis may actually occur more frequently than has previously been reported [38]. It is commonly detected inadvertently during surgery or from resected tissue specimens [35, 39], and the number of such metastases is reported to be 1 to 2 [32–34,36–40,]. Until now, lingual lymph nodes have not been mentioned in any level system of lymph node classification [37, 39]. Because ND generally does not include the lingual lymph nodes [36, 37, 39], there is some risk of tumor recurrence [37, 38]. Metastatic cells can spread from the primary tumor and reside in the lingual lymph nodes, but in most cases they move directly to the cervical lymph nodes [39]. When Leemans et al. [40] compared the results of transoral excision of the primary tumor with discontinuous ND with the results of in-continuity ND of the primary tumor and cervical lymph nodes in T2 oral cancer, they found a significantly worse rate of neck recurrence in the discontinuous ND group and regional recurrence was in the submandibular area in all instances. Thus, recurrence may have been due to remnant tumor cells left behind in the tissues between the primary site and the neck [40]. In-continuity resection with reconstruction, however, had a negative impact on both oral form and function.

Preoperative detection of lingual lymph nodes is difficult because of their small size and close proximity to the primary lesion [33, 39]; however, metastasis to the lingual lymph nodes could be an important prognostic factor [31, 32]. Therefore, additional imaging methods are required to detect this form of metastasis. Preoperative SLN mapping of N0 early oral cancer has commonly been performed using lymphoscintigraphy and/or SPECT/CT, with a reported identification rate of 95 to 100% [9, 11–13]. Nevertheless, SLN mapping using RI may be difficult for detecting these lingual lymph nodes because of their proximity to the primary tumor [31]. In contrast, CTL could clearly, and in 3D, identify lingual lymph nodes as well as cervical lymph nodes, including surrounding anatomical structures. Furthermore, CTL can detect SLN without RI and can be performed at many hospitals with conventional CT scanners. However, CTL cannot be performed for oral cancer patients with iodine allergy to iodinated contrast medium. Moreover, there were several limitations in this study such as the retrospective design and the small number of patients included. Therefore, further prospective studies involving a larger number of patients is required to assess protocol of CTL in SLN mapping of clinically N0 early oral cancer.

## Conclusion

CTL could facilitate the detection of SLNs in clinically N0 early oral cancer. The optimal timing of CT scanning was both 2 and 5 min after the administration of the contrast medium.

## Supplementary information

**Additional file 1: Table S1.** Patient characteristics.

## Data Availability

The datasets used and/or analyzed during the current study are available from the corresponding author on reasonable request.

## Abbreviations

3D: three-dimensional
BMI: body mass index
CT: computed tomography
CTL: computed tomography lymphography
FDG-PET/CT: 18F-fluorodeoxyglucose positron emission tomography/computed tomography
ICG: indocyanine green
MRI: magnetic resonance imaging
ND: neck dissection
RI: radioisotope
SLN: sentinel lymph node
SLNB: sentinel lymph node biopsy
US: ultrasonography
