# Supplementary material for: CT lymphography for sentinel lymph node mapping of clinically N0 early oral cancer
Source: Cancer Imaging. 2019 Nov 12;19:72. doi: 10.1186/s40644-019-0258-9 (PMC6852886; doi:10.1186/s40644-019-0258-9)
Supplement: Supplementary file 1 — Additional file 1: Table S1. Patient characteristics. [file 40644_2019_258_MOESM1_ESM.docx]

Table S1. Patient characteristics

| Patient code | Age (years) /Sex | BMI | Primary site | Clinical T classification |
| --- | --- | --- | --- | --- |
| A | 80/M | 25.8 | Tongue | 2 |
| B | 62/F | 19.0 | Tongue | 1 |
| C | 78/M | 23.4 | Floor of mouth | 1 |
| D | 45/F | 19.3 | Tongue | 1 |
| E | 54/F | 32.0 | Tongue | 1 |
| F | 47/F | 26.2 | Tongue | 1 |
| G | 74/M | 21.5 | Tongue | 2 |
| H | 47/M | 23.9 | Tongue | 2 |
| I | 70/M | 25.6 | Floor of mouth | 1 |
| J | 86/M | 18.0 | Tongue | 1 |
| K | 67/M | 17.5 | Tongue | 2 |
| L | 52/M | 29.4 | Tongue | 1 |
| M | 78/M | 23.1 | Tongue | 1 |
| N | 47/M | 29.0 | Tongue | 1 |
| O | 62/F | 21.1 | Tongue | 1 |
| P | 78/M | 23.1 | Tongue | 1 |
| Q | 65/F | 20.5 | Tongue | 1 |
| R | 77/F | 19.1 | Tongue | 1 |
| S | 23/F | 21.7 | Tongue | 1 |
| T | 71/M | 22.7 | Tongue | 2 |

BMI, body mass index; F, female; M, male
